# Dosing challenges for chemotherapy and immunotherapy in congenital achondroplasia: a case report and literature review

**DOI:** 10.1007/s00280-026-04932-7

**Published:** 2026-08-03

**Authors:** Karolijn W. M. Groot Beumer, E. M. J. Durlinger, S. W. J. Zielhuis, R. ter Heine, A. D. R. Huitema, J. J. M. A. Hendrikx

**Affiliations:** 1 Department of Clinical Pharmacology, Division of Medical Oncology, The Netherlands Cancer Institute, Amsterdam, The Netherlands; 2 Department of Thoracic Oncology, The Netherlands Cancer Institute, Amsterdam, The Netherlands; 3 Department of Pharmacy & Pharmacology, The Netherlands Cancer Institute, Amsterdam, The Netherlands; 4 Department of Pharmacy, Research Institute for Medical Innovation, Radboudumc, Nijmegen, The Netherlands; 5 Department of Clinical Pharmacy, University Medical Center Utrecht, Utrecht University, Utrecht, The Netherlands; 6 Princess Maxima Centre for Paediatric Oncology, Utrecht, The Netherlands; 7 Department of Nuclear Medicine, The Netherlands Cancer Institute, Amsterdam, The Netherlands

**Keywords:** Case report, NSCLC, Achondroplasia, Chemotherapy, Immunotherapy, Therapeutic drug monitoring

## Abstract

**Introduction:**

Currently, no established guidelines exist for dosing chemotherapy or immunotherapy in patients with achondroplasia.

**Case:**

A 69-year old female with congenital achondroplasia was diagnosed with stage IV non-small cell lung carcinoma type adenocarcinoma, with high Tumor Mutational Burden and 20% programmed death-ligand 1 expression. The patient received carboplatin dose based on renal function utilizing measured creatinine clearance, pemetrexed based on body surface area and pembrolizumab at a fixed dose of 100mg for body weight <65 kg. The doses of carboplatin and pemetrexed were adjusted after the first cycle based on therapeutic drug monitoring (TDM). The treatment was generally well tolerated, with the exception of grade 2 neutropenia, which resolved after a one-week delay of the third treatment cycle. A favorable clinical response was achieved after four treatment cycles.

**Conclusion:**

Given the uncertainty regarding the accuracy of dosing algorithms in patients with congenital achondroplasia, TDM may support dose optimization and attainment of adequate drug exposure.

## Introduction

Congenital achondroplasia is a variant of short-limbed dwarfism caused by a mutation in the fibroblast growth factor receptor 3 (FGFR3) gene [1, 2]. It is a rare condition, affecting roughly 4.6 per 100,000 live births [3], and currently, there are no established guidelines for dosing chemotherapy and immunotherapy in patients with this condition. The combination of carboplatin, pemetrexed and pembrolizumab is a widely used first-line regimen for advanced non-squamous non-small cell lung cancer (NSCLC) without targetable driver mutations, as established in the KEYNOTE-189 trial [4]. Carboplatin is dosed based on renal function, pemetrexed is dosed according to body surface area (BSA), and pembrolizumab is administered as a fixed dose or on mg/kg basis [5–7]. In patients with congenital achondroplasia, however, the accuracy of these dosing algorithms remains uncertain. Specifically, the applicability of BSA-based formulas in this population has not been established, and the Chronic Kidney Disease Epidemiology Collaboration (CKD-EPI) formula has not been validated for estimating kidney function in these patients due to uncertainty regarding BSA estimation and the CKD-EPI formula itself [8, 9]. Moreover, the validity of BSA-based dosing of a renally cleared drug such as pemetrexed is questionable. We present a case of congenital achondroplasia in which therapeutic drug monitoring (TDM) was applied to guide dose optimization of carboplatin, pemetrexed and pembrolizumab in the treatment of NSCLC.

## Case presentation

A 69-year-old female with congenital achondroplasia was referred to a pulmonologist for evaluation of dyspnea and a new abnormality on chest radiography. The patient reported coughing, weight loss and has a smoking history of 26 pack-years. A computed tomography (CT) scan of the thorax showed a mass of 4.3 centimeters in the left lower lobe of the lung and bilateral solid and ground-glass lung lesions suspicious for metastases. The patient subsequently underwent a bronchoscopy to obtain biopsies from the left lower lung lobe. Histopathological analysis confirmed the diagnosis of stage IV NSCLC type adenocarcinoma. Whole genome sequencing (WGS) showed no driver mutations, a smoking signature and high Tumor Mutational Burden (TMB) of 51.5 variants per megabase (Mb). Programmed death-ligand 1 (PDL-1) was positive in 20% of the tumor cells. Positron Emission Tomography-CT (PET-CT) demonstrated a hypermetabolic tumor in the left lower lobe of the lung and several fluorodeoxyglucose (FDG)-avid lesions in the right lung, a bone metastasis in the left clavicle, an FDG-avid lymph node in the lower right paratracheal region and at the level of the liver hilum. Considering the tumor characteristics, the proposed treatment strategy was to initiate chemo-immunotherapy with carboplatin, pemetrexed, and pembrolizumab. Laboratory tests showed normal hematology, normal liver function, serum creatinine of 21 µmol/l and estimated glomerular filtration rate (eGFR) of > 90 mL/min/1.73m2 based on the CKD-EPI formula. The patient had a height of 113 cm and a weight of 41.0 kg. Her BSA, determined from her measured height and weight using the DuBois and DuBois formula [10], was 1,07 m^2^. Given the potential inaccuracy of serum creatinine-based eGFR in patients with achondroplasia, 24-hour urine was collected and showed an adequate creatinine clearance of 89 mL/min. The creatinine clearance is 10–20% higher than the glomerular filtration rate because it represents the sum of passive filtration and active tubular secretion of creatinine [11]. The eGFR based on serum cystatin C and calculated using the CKD-EPI-Cys formula was > 90 mL/min/1.73m2. A dosing approach using BSA and eGFR formulas was pragmatically applied given the absence of validated alternatives for patients with achondroplasia. Standard supplementation with folic acid, vitamin B12 and dexamethasone was initiated according to the pemetrexed drug label.

During the first treatment cycle, the patient received carboplatin 570 mg, dosed to a target area under the curve (AUC) of 5 mg*min/mL using the Calvert formula, based on a measured creatinine clearance of 89 mL/min. The patient also received pemetrexed 535 mg (500 mg/m2) and pembrolizumab at a fixed dose of 100 mg for body weight < 65 kg [12, 13]. The pemetrexed dose was based on BSA due to the lack of better dosing strategies in patients with achondroplasia. However, in this context, BSA is used as a surrogate for drug clearance, and it is highly questionable whether BSA is an appropriate parameter for dosing of the renally cleared drug pemetrexed in patients with achondroplasia. The pembrolizumab dose was based on internal protocols and given as a fixed dose rather than a body-size-based dose [13]. The possible difference in volume of distribution of pembrolizumab in patients with congenital achondroplasia was anticipated to be not clinically relevant due to the wide therapeutic window of pembrolizumab [12]. The treatment cycles were administered every three weeks. Blood samples to measure drug concentrations of carboplatin, pemetrexed and pembrolizumab were collected after pemetrexed infusion, after carboplatin infusion and 2, 4, and 6 h after pemetrexed infusion. All blood samples were collected from the arm opposite to the one receiving the chemo-immunotherapy infusion. Pemetrexed, pembrolizumab, and carboplatin concentrations were determined in plasma, serum, and ultrafiltrate, respectively, using validated assays [14–16]. The first cycle of treatment was well tolerated and no toxicity was observed.

The estimated AUC of carboplatin was approximately 6.7 mg*min/mL during the first cycle, which is higher than the target AUC of 5 mg*min/mL [Table 1]. Clearance and AUC were estimated based on measured concentrations using a Bayesian approach with a previously published population pharmacokinetic (popPK) model [17]. To achieve an AUC closer to the target, the dose was reduced from 570 mg to 430 mg during the second cycle. The estimated AUC of pemetrexed was 110 mg*h/L, which is lower than the normal population AUC of 164 mg*h/L [18, 19] [Table 1]. A popPK model was used for Bayesian estimation of pemetrexed clearance and subsequent AUC calculation [20]. The pemetrexed dose could be increased by 25% to achieve an exposure closer to the population exposure. As the first cycle was well tolerated, the pemetrexed dose was, therefore, increased from 535 mg to 670 mg. Pembrolizumab was administered again at the same dose of 100 mg. The concentration measured immediately prior to the second pembrolizumab administration was ~ 7 µg/mL [Table 1], which is comparable to the mean trough concentration of 10.5 µg/mL (5th to 95th percentile 4.0–19.1 µg/mL) observed after the first dose of pembrolizumab 2 mg/kg every 3 weeks in patients with a body weight below 50 kg [21]. However, as the second treatment cycle was delayed by one week, the concentration was measured after a 4-week interval and does not represent a true trough concentration; the actual 3-week trough concentration is therefore likely higher and closer to the mean.

**Table 1:** Schematic overview of the dose and estimated AUC of carboplatin and pemetrexed during the first and second treatment cycles. The pembrolizumab doses and trough concentration after the first treatment cycle are shown.

	Dose first treatment cycle	Estimated AUC	Trough concentration	Dose second treatment cycle	Estimated AUC
Carboplatin	570 mg	6.7 mg*min/mL	N/A	430 mg	5.6 mg*min/mL
Pemetrexed	535 mg	110 mg*h/L	N/A	670 mg	164 mg*h/L
Pembrolizumab	100 mg	N/A	~ 7 µg/mL**	100 mg	N/A

** Measured prior to the second pembrolizumab administration (4-week interval); not a true trough concentration (3-week interval). *AUC: Area under the curve*

The second cycle was delayed by one week because the patient was unable to come to the hospital for logistical reasons. Pharmacokinetic assessments were performed again during the second treatment cycle. The estimated carboplatin AUC was approximately 5.6 mg*min/mL, which, while still exceeding the target AUC of 5 mg*min/mL, was closer to the target AUC of 5 mg*min/mL [Table 1]. Following dose escalation, the estimated pemetrexed AUC was 164 mg*h/L, which corresponds exactly to the population mean reported for pemetrexed therapy [Table 1] [19]. After the second treatment cycle, the only observed toxicities were grade 1 fatigue and grade 2 neutropenia according to the Common Terminology Criteria for Adverse Events (CTCAE) version 6.0. The neutropenia resolved without intervention, with only a one-week delay of the third cycle. As the treatment was overall well tolerated during cycle 2, no further dose adjustments were made for the third treatment cycle. At response evaluation after two treatment cycles, the patient had stable disease: reduction of the left lower lobe mass, the nodules of the right upper and lower lobes and FDG-avid lymph node, with the other lesions unchanged. Stable disease was maintained after four treatment cycles.

## Discussion

The selection of the right dose of therapy is challenging in the treatment of cancer. Patient-specific parameters are used to determine the appropriate dose to optimize efficacy while minimizing toxicity. Most cytotoxic therapies are dosed based on BSA, whereas monoclonal antibodies are often dosed based on body weight (mg/kg) or a fixed dose [22]. Nonetheless, these dosing strategies are based on studies in patients with normal body habitus and are expected to be inaccurate in individuals with atypical body composition. The American Society of Clinical Oncology (ASCO) guidelines recommend weight-based cytotoxic chemotherapy doses in obese patients with cancer [23]. In addition, standard approved doses of immunotherapy and targeted therapies are recommended in obese patients, as no evidence supports dose adjustment based solely on BMI [23]. However, apart from a single case report on chemotherapy dosing in a patient with achondroplastic dwarfism, very limited literature is available on the use of regular dosing formulas for dosing of chemotherapy and immunotherapy in patients with achondroplasia [24]. Consequently, determining appropriate dosing in this patient group is challenging, especially for drugs with a narrow therapeutic window such as cytotoxic chemotherapy.

Achondroplasia is the most common form of dwarfism, characterized by disproportionate body proportions, with relatively short limbs compared to the trunk, resulting in shorter stature [1]. Differences in body composition between individuals with achondroplasia and the general adult population, combined with the limited availability of literature, present challenges for determining appropriate dosing strategies in this population. In a study evaluating body composition in adult males with achondroplasia using dual X-ray absorptiometry (DEXA), participants demonstrated reduced fat-free mass and a higher body-fat mass as a whole-body measure compared with control subjects [25]. Altered body composition may contribute to differences in volume of distribution for some drugs (e.g. lipophilic drugs), potentially complicating the application of standard dosing strategies. Importantly, weight- and height-based dosing indices have not been validated in individuals with achondroplasia [26]. In addition, the popPK models used for the Bayesian estimation of carboplatin and pemetrexed clearance were developed in populations with standard body composition and are, therefore, not necessarily tailored to patients with achondroplasia [17, 20].

For the patient in our case report, the following dosing considerations were applied for the specific chemotherapy and immunotherapy treatments administered. Carboplatin is a platinum-based compound and is predominantly eliminated via renal excretion [17]. Therefore, the Calvert formula is applied to calculate an individualized dose based on the GFR, which is lower than the creatinine clearance, in order to attain the target AUC [5]. Since GFR estimation formulas have not been validated in patients with achondroplasia, renal function in our patient was assessed using multiple approaches, including cystatin C-based estimation and measured 24-hour creatinine clearance [27, 28]. These methods yielded comparable estimates of the renal function. The estimated carboplatin AUC during the first cycle was higher than expected, most likely because the patient’s renal clearance of carboplatin was lower than initially estimated based on the 24-hour urine collection [29]. A 25% dose reduction resulted in an AUC of 5.6 mg*min/mL during the second cycle [Table 1]; however, the model appears to underestimate the AUC, possibly because the volume of distribution is not adequately accounted for in this patient. In both cycles, the measured platinum concentration in the sample drawn right after carboplatin infusion was substantially higher than the model predicted concentration, indicating an overprediction of the volume of distribution. Although the model estimated the volume of distribution to be ~ 12 L during the first two treatment cycles, the actual volume of distribution is likely to be even lower. The estimated clearance and central volume of distribution estimates for our patient were 39–45% and 26–29% lower, respectively, than the corresponding population pharmacokinetic estimates [17]. Despite the relatively high carboplatin exposure, the dose was not adjusted for the third cycle because of uncertainties in the Bayesian estimation and the overall good tolerability of the treatment, with the exception of neutropenia requiring a one-week delay of the third cycle.

For pemetrexed, a cytostatic antifolate drug, toxicity is related to the time-above-threshold concentration [6]. This implies that prolonged high pemetrexed exposure due to reduced clearance from impaired renal function will lead to increased toxicity [6, 30]. Previously, in a study of patients with renal impairment in which the pemetrexed dose was adjusted according to renal function without folinic acid prophylaxis, all three patients developed severe grade III/IV myelotoxicity at only 50% of the intended dose, leading to premature termination of the study [6]. Folinic acid can antagonize pemetrexed toxicity and is mentioned in the label for treatment of severe toxicity, whereas standard prophylaxis during pemetrexed therapy consists of folic acid [31]. For this patient, pemetrexed was administered at the standard dose of 500 mg/m2 in the first cycle. Renal function was considered adequate at treatment initiation. However, there is uncertainty regarding the appropriateness of the formula used to calculate BSA in this patient, although this likely results in an underestimation. Therefore, the standard dosing strategy of 500 mg/m2 was considered safe, but potentially suboptimal with regard to efficacy, and drug concentrations were measured to optimize dosing. As expected, the estimated pemetrexed concentration during the first cycle was below the population mean. As pemetrexed AUC is a good predictor of toxicity in patients with adequate renal function [6], a dose increase was considered safe in this case. Therefore, the next pemetrexed dose was increased by 25% to approach the population mean AUC of 164 mg*h/L [19]. This dose was calculated using a formula for pemetrexed dosing based on renal function [30]. Carboplatin clearance was used as a measure for renal function, as the renal clearance of pemetrexed, similar to carboplatin, was likely lower in this patient than estimated from the 24-hour urine measurement. After the dose increase, the estimated AUC in the second cycle was 164 mg*h/L, equal to the population mean [Table 1]. The popPK model used for the Bayesian estimation of pemetrexed clearance includes renal function as a covariate [20]. As renal function could be estimated with reasonable accuracy using multiple approaches, the model is considered representative of the patient in this case. The individually model-predicted concentrations corresponded well with observed concentrations, indicating fit-for-purpose of the relatively rich sampling strategy, including informative samples shortly after end of infusion (informative for volume of distribution) and during the elimination phase. The model estimated the clearance to be 4.85 L/h and 4.08 L/h during the first and second treatment cycles, respectively. These estimates are comparable to the reported typical population value for pemetrexed clearance of 4.08 L/h [20].

In contrast to carboplatin and pemetrexed, pembrolizumab has a relatively wide therapeutic window [12]. The flat exposure–response relationship of pembrolizumab provides a rationale for using fixed dosing rather than weight-based (mg/kg) dosing [7, 12]. Moreover, pembrolizumab primarily distributes within the plasma and extracellular fluid, compartments whose volume does not increase proportionally with body weight [12]. As a result, fixed dosing reduces interpatient variability in exposure compared to weight-based dosing [12]. Given the wide therapeutic window of pembrolizumab, a fixed dose based on a body weight < 65 kg was considered an appropriate dosing approach for the patient described in our case. As expected, with this dosing regimen a trough concentration of ~ 7 µg/mL was measured four weeks after the first administration [Table 1], indicating adequate exposure within the population range and substantially higher than the threshold of linear kinetics of 0.68 µg/mL [32].

## Conclusion

Evidence and guideline recommendations regarding appropriate dosing of chemotherapy and immunotherapy regimens in patients with achondroplasia are limited. In this rare case of a patient with congenital achondroplasia treated with chemotherapy and immunotherapy, we demonstrate that therapeutic drug monitoring (TDM), together with clinical assessment, can support optimization of dosing. Overall, the patient tolerated the treatment regimen well and showed a favorable response after four treatment cycles. This case highlights that TDM is a valuable tool for achieving reasonable drug exposure in individual cases where the validity of standard dosing algorithms is uncertain, such as patients with altered body composition. However, further development of pharmacokinetic models is needed to better characterize drug exposure in these patients, as current models are often not representative of individuals with atypical body composition.

## Data Availability

No datasets were generated or analysed during the current study.
